# Growth and Essential Carotenoid Micronutrients in *Lemna gibba* as a Function of Growth Light Intensity

**DOI:** 10.3389/fpls.2020.00480

**Published:** 2020-05-07

**Authors:** Jared J. Stewart, William W. Adams, Christine M. Escobar, Marina López-Pozo, Barbara Demmig-Adams

**Affiliations:** ^1^Department of Ecology and Evolutionary Biology, University of Colorado, Boulder, CO, United States; ^2^Aerospace Engineering Sciences, University of Colorado, Boulder, CO, United States; ^3^Space Lab Technologies, LLC, Boulder, CO, United States

**Keywords:** co-optimization, duckweed, energy dissipation, human nutrition, photoprotection, photosynthesis, spaceflight, zeaxanthin

## Abstract

Duckweed is a promising food crop with multiple benefits for space applications. Fresh duckweed could deliver synergistically acting essential antioxidant nutrients to a crew – but only if growth conditions provide the plant with the right cues to trigger antioxidant formation. We grew *Lemna gibba* under continuous growth light ranging from low to very high intensities (photosynthetic photon flux densities = PPFDs) in order to investigate the effect on plant growth, photosynthesis, and level of carotenoid antioxidants that are essential human micronutrients. *Lemna gibba* achieved remarkably high growth rates under modest growth PPFD by virtue of superior light absorption resulting from minimal self-shading and high chlorophyll levels. Conversely, *L. gibba*’s growth rate remained high even under very high growth PPFDs. This notable ability of *L. gibba* to avoid inactivation of photosynthesis and diminished growth under very high growth PPFDs resulted from a combination of downregulation of chlorophyll synthesis and increased biochemical photoprotection that limited a build-up of excessive excitation energy. This biochemical photoprotection included accumulation of zeaxanthin (an essential human micronutrient) and high levels of zeaxanthin-catalyzed thermal energy dissipation of excess excitation. Compared to the light levels needed to saturate *L. gibba* photosynthesis and growth, higher light levels were thus required for strong induction of the essential antioxidant zeaxanthin. These results indicate a need for design of light protocols that achieve simultaneous optimization of plant yield, nutritional quality, and light-use efficiency to circumvent the fact that the light requirement to saturate plant growth is lower than that for production of high zeaxanthin levels. How this trade-off between light-use efficiency of growth and nutritional quality might be minimized or circumvented to co-optimize all desired features is discussed.

## Introduction

Self-sufficient life support systems for long-duration space exploration require reliable autonomous systems that use minimal amounts of expendables. Integration of photosynthetic organisms offers multi-functional regenerative life support in space, including production of food and oxygen, recycling of CO_2_ and other human waste, and recovery of water and nutrients. A good space crop should have a high growth rate, a high harvest index (be mostly edible), and high nutritional value, while requiring minimal resources such as occupied volume, water, and energy.

Duckweeds (family Lemnaceae) are small, floating aquatic plants that are 100% edible, nutritious, non-toxic, fast growing, and able to purify nutrient-rich wastewater (Leng, 1999; Appenroth et al., 2017, 2018; Chen et al., 2018; Iatrou et al., 2019; Sree et al., 2019). Duckweed grows continuously by propagation and is among the fastest-growing plants with respect to its doubling rate, e.g., doubling its biomass in as little as 1 to 3 days (Ziegler et al., 2015). Duckweed has, therefore, received attention as a potential crew food supplement or wastewater treatment for the human space program (Ward and Wilks, 1963; Wilks, 1964; Woverton and McDonald, 1977; Gale et al., 1989; Bluem and Paris, 2001; Polyakov et al., 2010). Moreover, Yuan and Xu (2017) found that simulated microgravity actually stimulated duckweed growth and called duckweed “one of the most attractive higher plants” for long-duration space life support.

To realize duckweed’s full potential, optimal growing conditions for high yields of nutritious food with the fewest spacecraft resources need to be defined in an environment relevant for space missions. Although duckweed has tremendous potential for both high productivity and nutritional value, duckweed can be expected to be subject to a universal trade-off in plant physiology. Energy-efficient plant biomass production comes at the cost of poor micronutrient quality of plant biomass (especially antioxidant vitamins) and *vice versa*. This trade-off is caused by the fact that a growth light intensity (photosynthetic photon flux density, PPFD) just enough to support maximal plant growth is not enough to induce high vitamin/antioxidant levels for principal reasons (Polutchko et al., 2015). Only exposure to *excess* PPFD, defined as more light than needed to saturate growth, prompts plants to accumulate protective antioxidants that prevent damage by excess light. However, such excess PPFD has the potential to negatively impact photosynthesis, and, by the above definition, represents input of more light than needed to maximize growth, which lowers the light-use efficiency of plant productivity. This fundamental link leads to a trade-off between light-use efficiency and antioxidant micronutrient content, the severity of which varies among plant species and growth conditions. Plant species vary in their tolerance of very low or very high growth PPFD and most species do not thrive equally well in deep shade and full sun (Adams et al., 2018). Shade-tolerant species, in particular, can exhibit lasting depressions in the efficiency of primary photochemistry (a phenomenon termed photoinhibition of photosynthesis) when grown under high PPFD (Adams et al., 2013, 2014, 2018). A major component of the response to growth under higher PPFD compared to low light is the increased synthesis of photoprotectors and antioxidants (Grace and Logan, 1996; Logan et al., 1996, 1998a,b).

Antioxidant metabolites can be categorized into a large group of water-soluble metabolites and a small set of water-insoluble, membrane-bound metabolites. We here focus on water-insoluble carotenoids (the xanthophylls zeaxanthin and lutein as well as β-carotene) that protect biological membranes (Demmig-Adams and Adams, 2013; Demmig-Adams et al., 2013; Polutchko et al., 2015). Deficiency in these antioxidants causes production of membrane break-down products that trigger chronic inflammation in humans as root causes of all major chronic diseases and disorders (Demmig-Adams and Adams, 2002, 2013). Ionizing radiation, a major challenge in space, produces oxidants and triggers chronic inflammation throughout the body (Azzam et al., 2012). Foods rich in zeaxanthin, lutein, and β-carotene were shown to protect against damage by ionizing radiation in a population of airline pilots (Yong et al., 2009; see also Asker et al., 2007). Zeaxanthin is the primary antioxidant photoprotector in leaves as well as the human eye and other tissues (Demmig-Adams and Adams, 2002, 2013), yet is much harder to come by in the diet than lutein or β-carotene. Lutein plays a secondary role in the protection of leaves and the human eye, yet is more abundant in plant-based food (Demmig-Adams and Adams, 2002, 2013; Polutchko et al., 2015). Several plant regulatory mechanisms ensure that zeaxanthin is formed in leaves only under excess light and is removed quickly upon return to non-excessive light levels (Demmig-Adams et al., 1996b). β-carotene can also serve as an antioxidant in both plants (Telfer, 2014) and animals (Stahl and Sies, 2012) and is, furthermore, the precursor (pro-vitamin A) in the synthesis of vitamin A that is converted to the light-absorbing (retinal) component of the human eye (Polutchko et al., 2015). Zeaxanthin also has a second role in the direct protection of membrane lipids and acts synergistically with vitamin E in this role (Wrona et al., 2003, 2004; Schneider, 2005).

Unlike most plants, duckweed contains all amino acids essential for humans and has a fat composition with high levels of beneficial essential fatty acids (Appenroth et al., 1982, 2017; Yan et al., 2013). Duckweeds also produce high levels of other essential micronutrients for humans that have to be supplied by the human diet and include the synergistically acting antioxidant carotenoids zeaxanthin, lutein, and β-carotene and the antioxidant vitamins C (ascorbic acid) and E (especially α-tocopherol essential to human health) (Appenroth et al., 2017, 2018). Dietary zeaxanthin, in particular, increases visual acuity, protects the human eye against damage from intense light (prevents cataracts and blindness), and combats inflammation throughout the body (Demmig-Adams and Adams, 2013). A good dietary supply of zeaxanthin is thus critical for astronauts exposed to dangerous levels of damaging radiation. However, uptake of these micronutrients from supplements can be poor in the human gut, particularly for the water-insoluble vitamins A, E, and carotenoids that are best taken up from a food matrix (Tran and Demmig-Adams, 2007; Demmig-Adams and Adams, 2013; Rodriguez-Casado, 2016). Plants produce these antioxidants for their own protection against damaging radiation that contributes to oxidant production. In the case of zeaxanthin, leaves accumulate a zeaxanthin precursor (violaxanthin) and convert the latter to zeaxanthin only under exposure to more light than plants can use for growth (Demmig-Adams and Adams, 2002). Fresh duckweed can deliver a balanced mix of these synergistically acting essential nutrients to a crew – but only if growth conditions provide the plant with the right cues to trigger antioxidant formation. This critical link between plant growth conditions and leaf antioxidant content is the focus of the present study. We grew *Lemna gibba* over a wide range of growth PPFDs (with continuous light, i.e., 24 h per day), ranging from low (100 μmol m^–2^ s^–1^) to very high (700 μmol m^–2^ s^–1^), in order to investigate the effect of growth light intensity on growth rate and the production of essential human micronutrient carotenoids.

## Materials and Methods

### Plant Material and Growth Conditions

*Lemna gibba* L. 7741 (G3), obtained from Rutgers Duckweed Stock Cooperative^1^, was used for all experiments. Plants were grown in 150 × 75 mm PYREX Crystallizing Dishes (Corning Inc., Corning, NY, United States) containing 1000 mL of Schenk and Hildebrandt Medium (bioWORLD, Dublin, OH, United States; Schenk and Hildebrandt, 1972) at a concentration of 1.6 g L^–1^ and a pH of 5.5 (adjusted via 1% [w/v] KOH). To minimize microbial contamination, media were prepared with recently boiled water prior to each transfer of fronds to new dishes. The volume of each dish was monitored every day and adjusted with recently boiled water to compensate for evaporative water loss. Stock cultures were maintained under a constant PPFD of 50 μmol m^–2^ s^–1^ supplied via fluorescent (F72T12/CW/HO; Philips, Somerset, NJ, United States) and incandescent (100W, 130V; EiKO, Shawnee, KS, United States) bulbs and an air, water, and frond temperature of 25°C in a Conviron PGR15 growth chamber (Controlled Environments Ltd., Winnipeg, MB, Canada). During cultivation, a subset of approximately 20 fronds from each dish were transferred using sterile inoculation loops (2865, Globe Scientific, Mahwah, NJ, United States) to fresh media in clean dishes at least once per week (typically after 4 days).

**FIGURE 1:**
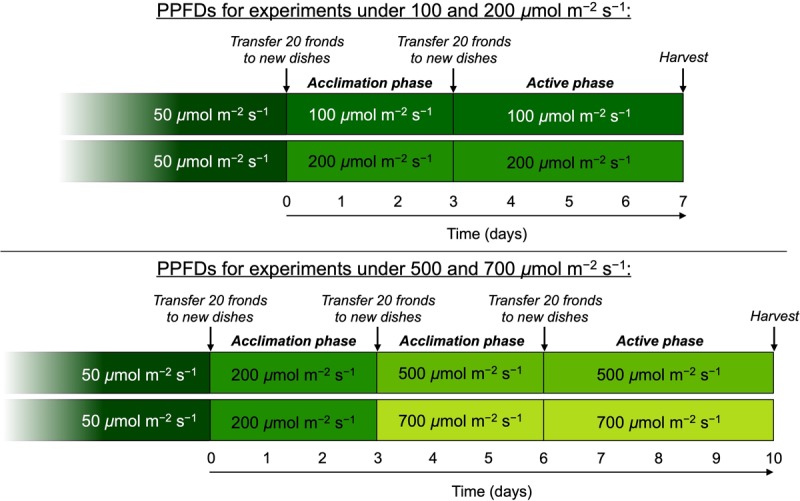
Schematic depicting the protocol for growth of *L. gibba* under different photosynthetic photon flux densities (PPFDs). All plants were initially cultivated under 50 μmol photons m^–2^ s^–1^ and then transferred to either 100 or 200 μmol photons m^–2^ s^–1^. Following 3 days of acclimation, growth rate for these two PPFDs was monitored for 4 days after which fronds were sampled for the various features characterized. For the higher growth light intensities, additional fronds were transferred from 200 μmol photons m^–2^ s^–1^ to each of the PPFDs where each experienced a 3-day acclimation period followed by 4 days of characterized growth and sampling for the other parameters.

Experimental plants were grown under four light intensities, with PPFDs of 100, 200, 500, and 700 μmol m^–2^ s^–1^. All plants were grown under continuous light at ambient CO_2_ concentrations (approximately 400 ppm in Boulder, CO) and a water and frond temperature of 25°C since duckweed growth is enhanced under continuous light versus shorter photoperiods (Yin et al., 2015; Liu et al., 2018). For experiments, plants were transferred to, and cultivated under, each respective final growth PPFD for 7 days (Figure 1). For growth experiments under 100 and 200 μmol photons m^–2^ s^–1^, plants were acclimated to these PPFDs for 3 days, followed by 4 days of experimentation with full monitoring of growth rates and other parameters (Figure 1). For experiments under growth PPFDs of 500 and 700 μmol m^–2^ s^–1^, plants were transferred to 200 μmol m^–2^ s^–1^ for 3 days before transfer to the respective final experimental PPFDs (Figure 1). At the time of each transfer to a new growth PPFD as well as at the start of the 4-day active phase of each experiment subsequent to 3 days of acclimation, frond density was reset to 20 fronds per dish by transferring only 20 fronds to fresh medium in a new dish. Growth experiments were conducted successively in the same growth chamber with three replicate dishes illuminated by three identical custom-built panels of white LEDs (C503C-WAN; CREE Inc., Durham, NC, United States; for details on LED features, see Burch, 2016). Temperature of media was monitored during the 3-day acclimation phase using alcohol-sterilized thermometers, and frond temperature kept constant at 25°C. Under the higher growth PPFDs, air temperature was lowered from 25°C until temperature of the media was maintained at 25°C for 24 h. Resulting adjusted air temperatures were 24 and 20°C for growth PPFDs of 500 and 700 μmol m^–2^ s^–1^, respectively. Since floating duckweed has unlimited access to water and its stomates are inactive, i.e., are kept fully open over a wide range of conditions (McLaren and Smith, 1976), any differences in transpiration rate at these different air temperatures would not be expected to impact the parameters assessed in this study. Immediately prior to both acclimation and onset of experimental phases, a subset of approximately 20 fronds from each dish was transferred to fresh, filtered medium in clean dishes. Medium was filtered through Fisherbrand P5 filter paper (Fisher Scientific, Hampton, NH, United States) prior to the acclimation phase and 0.22-μm Millipak-20 filters (Millipore, Billerica, MA, United States) prior to the active test phase.

### Growth Rate and Photon Flux

**FIGURE 2:**
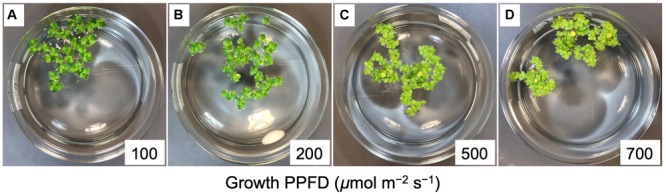
Images of crystallizing dishes with *L. gibba* fronds following a 4-day growth experiment at each of the four different PPFDs investigated, which correspond to the numbers (100 to 700) in each of the panels **A** through **D**. Each dish started with approximately 20 fronds. Mother fronds were slightly darker than daughter fronds. Note the greater number of fronds per colony and greater proliferation of roots at the higher growth PPFDs.

Once per day during the 4-day active phase of each experiment, digital photographic images of each dish were taken from directly above and perpendicular to the surface of the media (see images in Figure 2). Frond area was measured using MATLAB Image Processing Toolbox (MathWorks, Natick, MA, United States) by first selecting the pixels containing the water surface inside the dish (i.e., those masking the water surface) and then selecting pixels inside the masked area containing green fronds with a color thresholding algorithm. Frond area was calculated as the percentage of total water surface containing fronds times the known water surface area of the crystallizing dish (145-mm inner diameter).

Relative growth rate was calculated as the difference in ln-adjusted frond areas divided by the time elapsed (in days) between the two measurements (see, e.g., Hunt, 1990), as shown in the equation below (where **FA_4_** and **FA_0_** are the frond areas at the end and beginning of the 4-day experiment, respectively, and **t_4_** is the time elapsed between these two measurements):

$$R\,e\,l\,a\,t\,i\,v\,e\,g\,r\,o\,w\,t\,h\,r\,a\,t\,e=\frac{l\,n\,\left(F\,A_{4}\right)-l\,n\,\left(F\,A_{0}\right)}{t_{4}}$$

Relative growth rate (**RGR**) and initial frond area (**FA_0_**) for each dish were then used to construct the following generalized relationship between frond area (in m^2^) and time elapsed (**t**):

$$F\,r\,o\,n\,d\,a\,r\,e\,a\,\left(t\right)=F\,A_{0}\times e^{R\,G\,R\times t}$$

For each PPFD (in μmol photons m^–2^ s^–1^), the rate at which plants within each dish received photons scaled proportionally with their frond area. Thus, the following generalized relationship was used for photon flux for plants within each dish (in mol day^–1^ with conversion factors of 0.000001 mol μmol^–1^ and 86,400 s day^–1^) and time:

$$P\,h\,o\,t\,o\,n\,f\,l\,u\,x\,\left(t\right)=P\,P\,F\,D\times 0.0864\times F\,A_{0}\times e^{R\,G\,R\times t}$$

Number of photons incident on the plant surface within a dish during the 4-day experiments was estimated using the following equation:

$$P\,h\,o\,t\,o\,n\,s\,r\,e\,c\,e\,i\,v\,e\,d\,\left(t_{4}\right)=\int_{0}^{t_{4}}P\,h\,o\,t\,o\,n\,f\,l\,u\,x\,\left(t\right)\,d\,t$$

Light-use efficiency (as m^2^ frond area produced per mol photons received during the experiment) was calculated as:

$$L\,i\,g\,h\,t\,\text{-}\,u\,s\,e\,e\,f\,f\,i\,c\,i\,e\,n\,c\,y=\frac{F\,A_{4}-F\,A_{0}}{P\,h\,o\,t\,o\,n\,s\,r\,e\,c\,e\,i\,v\,e\,d\,\left(t_{4}\right)}$$

Frond dry mass was quantified with an A-160 balance (Denver Instruments Company, Denver, CO, United States) from fronds that had been dried at 70°C for 7 days, and frond area of each sample quantified with ImageJ software (Schneider et al., 2012) from images taken before samples were placed in the drying oven.

### Photosynthetic Capacity

Photosynthetic capacity was determined as light- and CO_2_-saturated rate of net photosynthetic oxygen evolution with oxygen electrode systems (Hansatech Instruments Ltd., Norfolk, United Kingdom; see Delieu and Walker, 1981) coupled with circulating water baths set to 25°C as previously described (Dumlao et al., 2012). For plants grown under PPFDs of 100 to 500 μmol m^–2^ s^–1^, oxygen evolution was measured from fronds of a known area after 5-min exposures to 500, 1000, and 1500 μmol photons m^–2^ s^–1^. Plants grown under 700 μmol photons m^–2^ s^–1^ were assayed at PPFDs of 1000, 1500, and 2000 μmol m^–2^ s^–1^. Frond areas were quantified from images of samples taken either immediately before or after each measurement using ImageJ.

### Chlorophyll Fluorescence

**FIGURE 3:**
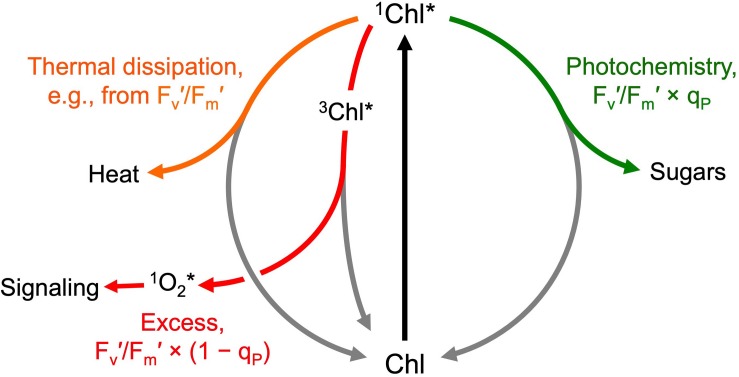
Allocation of absorbed photons (after exciting chlorophyll from the ground state, Chl, to the singlet excited state, ^1^Chl*) to photochemistry (available for photosynthesis and growth; green arrow), photoprotective thermal energy dissipation (facilitated via zeaxanthin; orange arrow), and the remaining excess excitation energy removed neither via photosynthesis nor thermal energy dissipation (red arrow). The excess excitation energy leaves the system via conversion of singlet excited chlorophyll (^1^Chl*) to triplet excited chlorophyll (^3^Chl*) and subsequent transfer of the excitation energy to oxygen resulting in the formation of singlet excited oxygen (^1^O_2_*) that can oxidize polyunsaturated membrane lipids to gene regulators (signaling) or, in large amounts, lead to membrane damage. The allocation of photons to these different fates under a given condition can be estimated from chlorophyll fluorescence emission (see text for further explanation of the parameters shown).

Chlorophyll fluorescence was measured with a PAM-101 chlorophyll fluorometer (Walz, Effeltrich, Germany) to estimate allocation of absorbed light to photosynthesis versus photoprotective thermal energy dissipation as well as excitation energy not removed via either photosynthesis or energy dissipation as previously described (Figure 3; Genty et al., 1989; Demmig-Adams et al., 1996a; Adams and Demmig-Adams, 2004; Logan et al., 2014). Maximal chlorophyll fluorescence yield (with all photosystem II [PSII] centers in the reduced state and unavailable to perform photochemistry) was determined after 5 min of dark incubation (F_m_) and after 5 min of exposure to PPFDs corresponding to the respective growth PPFDs (F_m_′). Steady-state fluorescence under the respective growth PPFD (F) was determined immediately before measurement of F_m_′. The yield of minimal chlorophyll fluorescence (with all PSII centers in the open state ready to perform photochemistry) was determined after 5 min of dark incubation (F_o_) and upon brief darkening after steady-state fluorescence had been reached during exposure to actinic light corresponding to the respective growth PPFD (F_o_′). Variable fluorescence after dark incubation (F_v_) and after exposure to growth PPFD (F_v_′) were F_m_ – F_o_ and F_m_′ – F_o_′, respectively. Potential maximal PSII photochemical efficiency after discontinuation of light-dependent thermal energy dissipation activity is given by F_v_/F_m_ of darkened leaves (fronds darkened for 5 min either immediately upon removal from growth PPFD or after 30 min of recovery in low light of 10 μmol m^–2^ s^–1^). Maximal photochemical efficiency in the light (in the presence of light-dependent thermal energy dissipation) of those PSII centers that remain open at each growth PPFD is given by F_v_′/F_m_′. The fraction of PSII centers that are closed (reduced) under each PPFD is given by 1 – q_P_ = (F – F_o_′)/(F_m_′ – F_o_′), where q_P_ stands for photochemical quenching. Photochemical efficiency at the percentage of closed PSII centers under each respective growth PPFD is given by F_v_′/F_m_′ × q_P_. The latter efficiency determines the efficiency of photosynthetic electron transport. In leaves that do not exhibit photoinhibitory inactivation of photosynthesis (see section “Results”), the fraction of absorbed photons dissipated by photoprotective energy dissipation under each respective growth PPFD can be assessed as 0.8 – F_v_′/F_m_′ (see Demmig-Adams et al., 1996a). The fraction of absorbed photons used neither in photochemistry nor in thermal energy dissipation is given by F_v_′/F_m_′ × (1 – q_P_) (Demmig-Adams et al., 1996a).

### Chlorophylls, Carotenoids, and Starch

Chlorophylls *a* and *b*, β-carotene, lutein, zeaxanthin, antheraxanthin, violaxanthin, and neoxanthin levels were determined via high-performance liquid chromatography as previously described (Stewart et al., 2015). From each dish under each growth PPFD, two samples of approximately 10 fronds each were harvested for pigment analysis at the same time as samples of fronds were taken for chlorophyll fluorescence measurements, i.e., before and after 30 min of recovery under 10 μmol photons m^–2^ s^–1^. After removal of roots, fronds were imaged for area with ImageJ and frozen in liquid nitrogen. Pigment concentrations for each dish were expressed as average concentrations of the two samples harvested before and after 30 min of recovery for those pigments that do not change in concentration over 30 min, i.e., chlorophyll *a* and *b*, lutein, β-carotene, the sum of the xanthophyll cycle pigments violaxanthin + antheraxanthin + zeaxanthin (V + A + Z), and total carotenoids (lutein, β-carotene, V, A, Z, and neoxanthin). Zeaxanthin level was calculated separately for samples collected before versus after 30 min of recovery. The reported zeaxanthin levels are from samples collected before the recovery unless otherwise stated.

For qualitative assessment of starch content, fronds were cleared in 70% (v/v) ethanol, stained for 5 min with diluted iodine-potassium iodide solution (Lugol’s solution; Sigma-Aldrich, St. Louis, MO, United States), and immediately mounted and imaged with a high-resolution scanner (Perfection 3200 Photo; Epson America, Inc., Long Beach, CA, United States).

### Statistical Analyses

Statistically significant differences among growth PPFDs were determined via analysis of variance (one-way ANOVA) and *post hoc* Tukey–Kramer test for Honestly Significant Differences. Lines of best fit were obtained using non-linear models. Analyses were conducted with JMP Pro 15.0.0 (SAS Institute Inc., Cary, NC, United States). Sample sizes for each experiment was 3 dishes as also indicated in figure legends.

## Results

### Growth Rates and Light-Use Efficiency of Growth

**FIGURE 4:**
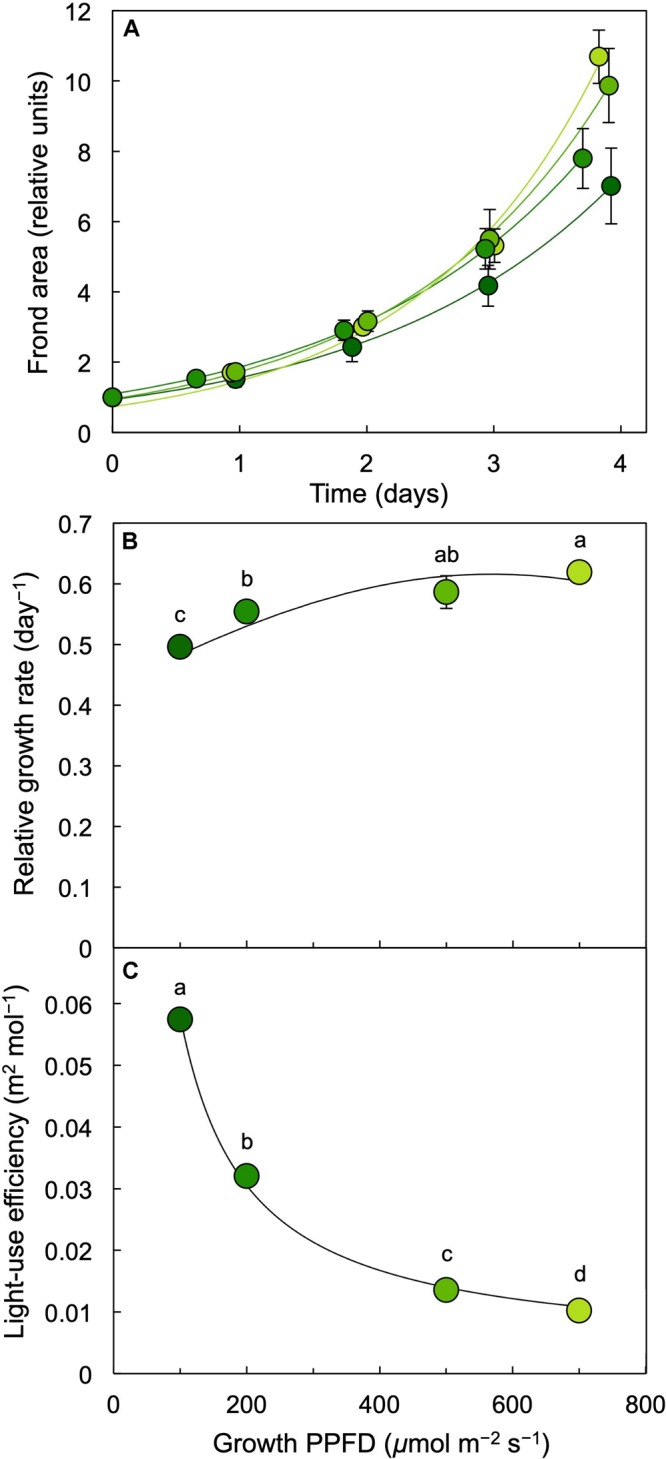
**(A)** Frond area accumulation (starting values normalized to 1) over a period of 4 days, **(B)** relative growth rate (difference in ln-adjusted frond areas per time elapsed) as a function of growth PPFD, and **(C)** light-use efficiency of *L. gibba* frond area production as a function of growth PPFD. The shades of green to yellow of the symbols correspond to the respective growth PPFDs (see Figure 1 and **B**,**C**), ranging from darker green (for 100 μmol m^–2^ s^–1^) to light green (for 700 μmol m^–2^ s^–1^). Mean values ± standard deviations, *n* = 3 for all growth PPFDs; different lower-case letters signify statistical differences at *P* < 0.05 via one-way analysis of variance and *post hoc* Tukey–Kramer HSD test.

Dishes of *L. gibba* were photographed (Figure 2) once per day over 4 days of growth under PPFDs ranging from 100 to 700 μmol photons m^–2^ s^–1^ of continuous light. These images indicate that a similar level of frond growth was maintained over this range of growth PPFDs (Figures 2, 4). Due to these similar trajectories of exponential growth, corresponding growth curves were largely overlapping (Figure 4A). Relative growth rates (difference in ln-adjusted frond areas per unit of time) were consequently also similar (Figure 4B). In other words, *L. gibba* achieved rather high growth rates at low growth PPFDs and maintained similar growth rates over a wide range of growth PPFDs. Due to the fact that a 7-fold increase in growth PPFD (from 100 to 700 μmol m^–2^ s^–1^) did not produce a great deal of additional growth, light-use efficiency of area production was maximal at the lowest growth PPFD and declined precipitously with increasing growth PPFD (Figure 4C). Specifically, a 25% greater relative growth rate at 700 (maximal RGR) versus 100 μmol photons m^–2^ s^–1^ came at the cost of a 600% greater input of light.

### Frond Content of Photosynthetic Pigments

**FIGURE 5:**
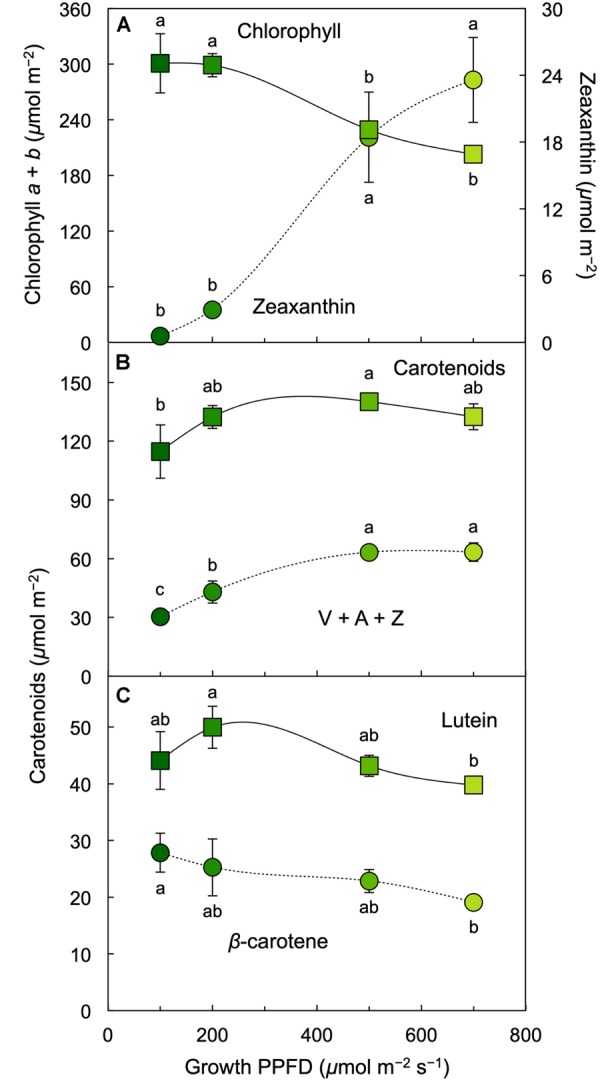
Levels of **(A)** chlorophyll *a* + *b* (squares) and zeaxanthin (circles), **(B)** carotenoids (sum of V, A, Z, lutein, β-carotene, and neoxanthin; squares) and the xanthophyll cycle pool (V + A + Z; circles), and **(C)** lutein (squares) and β-carotene (circles) on an *L. gibba* frond area basis as a function of growth PPFD. A, antheraxanthin; V, violaxanthin; Z, zeaxanthin. Mean values ± standard deviations, *n* = 3 for all growth PPFDs; different lower-case letters signify statistical differences at *P* < 0.05 via one-way analysis of variance and *post hoc* Tukey–Kramer HSD test.

Chlorophyll content on a frond area decreased at growth PPFDs between 200 and 700 μmol m^–2^ s^–1^ (Figures 2B–D, 5A). On a frond areas basis, total carotenoids increased with increasing growth PPFD and remained similar up to the highest growth PPFD. This pattern was accounted for by the trends of the constituent carotenoid fractions. While the total pool of the xanthophyll cycle pigments violaxanthin, antheraxanthin, and zeaxanthin (V + A + Z) showed an increasing trend with increasing growth PPFD, the levels of the individual carotenoids lutein and β-carotene declined between 200 and 700 μmol photons m^–2^ s^–1^ (Figures 5B,C). The only individual carotenoid that showed a unique, contrasting trend was zeaxanthin that was at negligible levels under the lowest growth PPFD and increased successively with each increase in growth PPFD between 200 and 700 μmol m^–2^ s^–1^ (Figure 5A).

**FIGURE 6:**
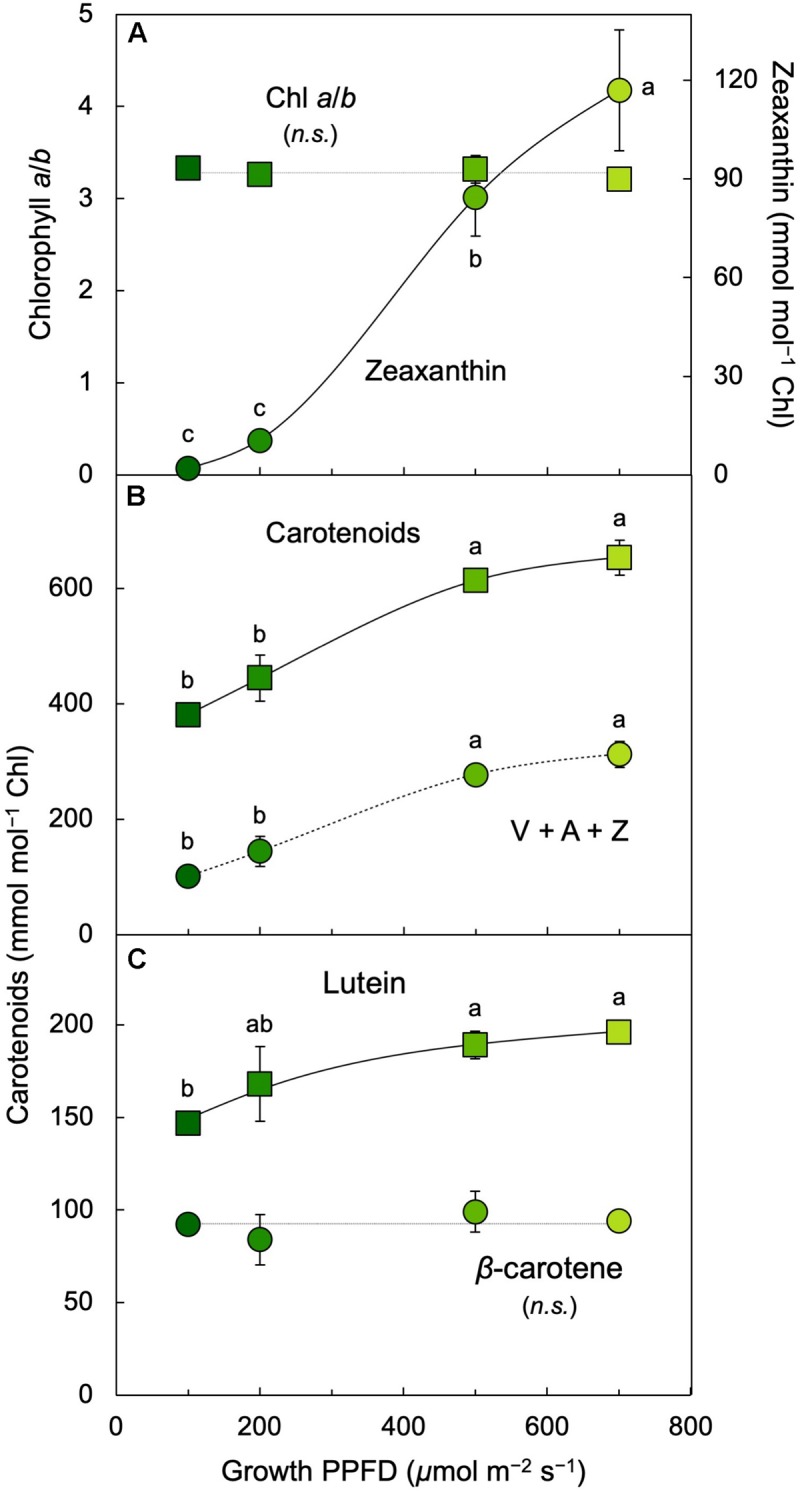
**(A)** Chlorophyll *a*/*b* ratio (squares) and zeaxanthin per chlorophyll (Chl; circles), **(B)** total carotenoids (sum of V, A, Z, lutein, β-carotene, and neoxanthin; squares) and xanthophyll cycle pool (V + A + Z; circles) per chlorophyll, and **(C)** lutein (squares) and β-carotene (circles) per chlorophyll in *L. gibba* as a function of growth PPFD. A, antheraxanthin; V, violaxanthin; Z, zeaxanthin. Mean values ± standard deviations, *n* = 3 for all growth PPFDs; different lower-case letters signify statistical differences at *P* < 0.05 via one-way analysis of variance and *post hoc* Tukey–Kramer HSD test (n.s., not significantly different).

The ratio of chlorophyll *a* (bound to all chlorophyll antennae) to chlorophyll *b* (bound only to the outer chlorophyll antennae that maximize light absorption under limited light levels) remained constant between 100 and 700 μmol photons m^–2^ s^–1^ (Figure 6A) whereas chlorophyll *a* + *b* levels declined (Figure 5A). All carotenoids increased relative to chlorophyll with increasing growth PPFD (Figures 6B,C). The increase of the total carotenoid pool was paralleled by an increase in the xanthophyll cycle pool (violaxanthin, antheraxanthin, and zeaxanthin; Figure 6B) and in lutein on a chlorophyll basis (Figure 6C). β-carotene relative to chlorophyll exhibited only a modest increase with increasing growth PPFD (Figure 6C). The carotenoid that exhibited the strongest increase with growth PPFD on a chlorophyll basis was, again, zeaxanthin (Figure 6A).

### Photosynthetic Capacity, Light-Use Efficiency, Energy Dissipation, and Excess Excitation

**FIGURE 7:**
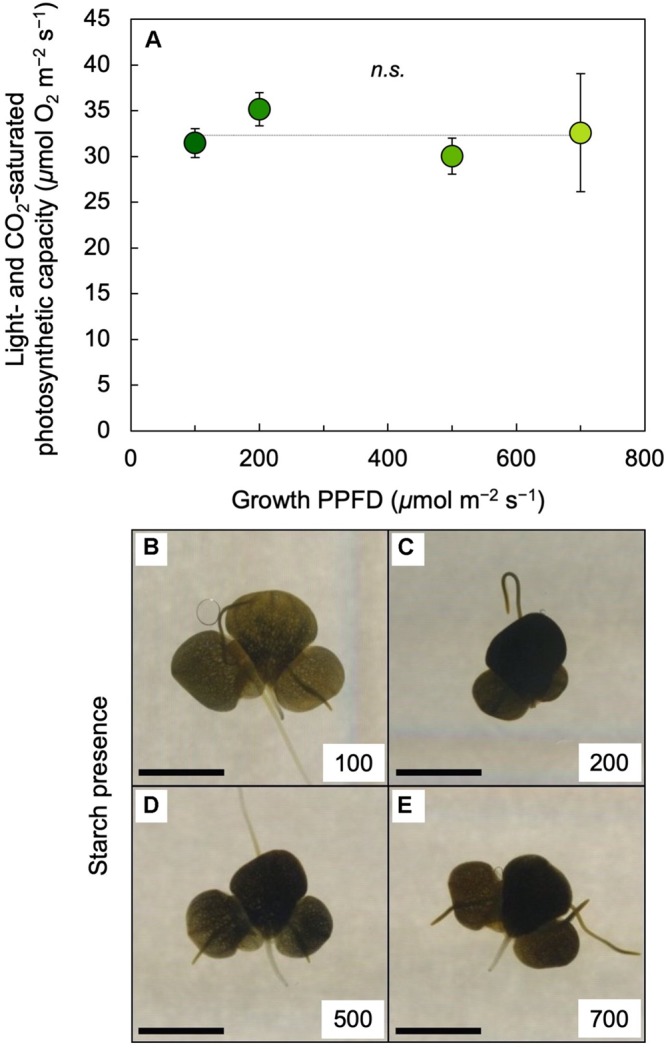
**(A)** Light- and CO_2_-saturated rate of oxygen evolution determined at 25°C and (**B** through **E**) iodine stain (as a qualitative test for starch presence) of *L. gibba* fronds as a function of growth PPFD. The numbers (100 to 700) in each of the panels **B** through **E** correspond to the PPFD (in μmol photons m^–2^ s^–1^) under which fronds were grown; the scale bars represent 5 mm. In **A**, mean values ± standard deviations, *n* = 3 for all growth PPFDs; n.s., not significantly different at *P* < 0.05 via one-way analysis of variance. Mean values ± standard deviations for frond dry mass per area (g m^–2^) were 26.4 ± 2.2, 37.4 ± 5.5, 37.9 ± 5.3, and 37.8 ± 3.8 for growth PPFDs of 100, 200, 500, and 700 μmol m^–2^ s^–1^, respectively (*n* = 3 for all growth PPFDs).

Light-and CO_2_-saturated photosynthetic capacity on a frond area basis was not significantly different between 100 and 700 μmol photons m^–2^ s^–1^ (Figure 7A) and neither was dry mass per leaf area (see legend of Figure 7). To probe whether the absence of a PPFD-dependent increase in photosynthetic capacity may be associated with starch accumulation, an iodine starch test was performed. This test indicated modest starch accumulation in fronds grown under 100 μmol photons m^–2^ s^–1^ (Figure 7B) and strong starch accumulation in fronds grown under PPFDs between 200 and 700 μmol m^–2^ s^–1^ (Figures 7C–E).

**FIGURE 8:**
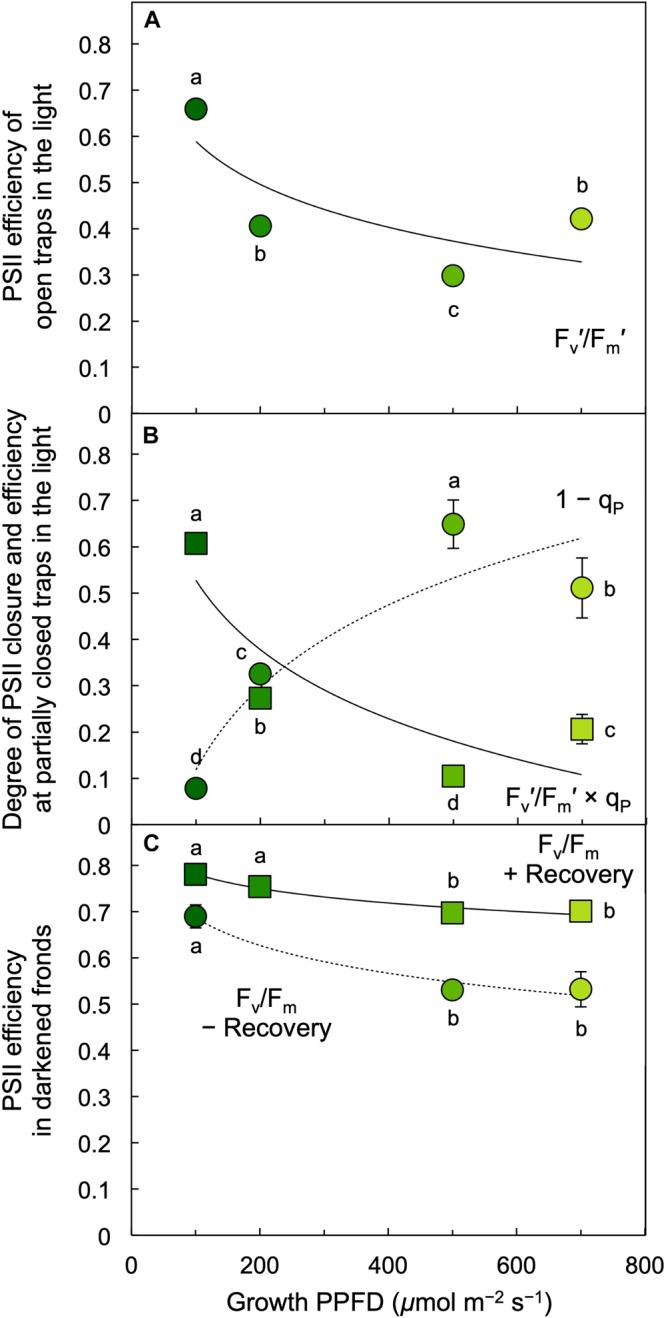
**(A)** Efficiency of open photosystem II (PSII) centers (traps) during exposure to each respective growth PPFD, **(B)** fraction of PSII reaction centers (traps) that are closed, 1 – q_P_ = (F – F_o_′)/(F_m_′ – F_o_′) (circles), and PSII efficiency at the degree of center closure (F_v_′/F_m_′ × q_P_; squares) under each respective growth PPFD, and **(C)** the efficiency of PSII in the dark (F_v_/F_m_) in *L. gibba* fronds grown under each respective PPFD. Dark F_v_/F_m_ was determined immediately upon removal of fronds from growth light conditions (– Recovery) and again after 30 min in low light (+ Recovery). F_m_′, maximal fluorescence under actinic light; F_v_′, variable fluorescence under actinic light (F_m_′ – minimal fluorescence F_o_′); PSII, photosystem II; q_P_, photochemical quenching. Mean values ± standard deviations, *n* = 3 for all growth PPFDs; different lower-case letters signify statistical differences at *P* < 0.05 via one-way analysis of variance and *post hoc* Tukey–Kramer HSD test.

**FIGURE 9:**
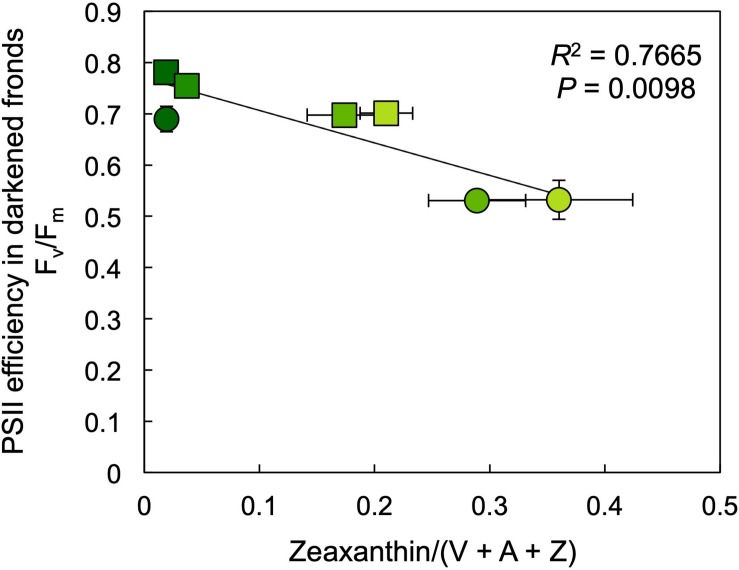
Relationship between the efficiency of open photosystem II (PSII) centers, F_v_/F_m_, and zeaxanthin content (as a fraction of the total xanthophyll cycle pool, violaxanthin [V], antheraxanthin [A], and zeaxanthin [Z]) in samples collected either immediately (circles) or after a recovery period of 30 min under a low PPFD of 10 μmol m^–2^ s^–1^ (squares) subsequent to removal from growth PPFDs. F_m_, maximal fluorescence in the dark; F_v_, variable fluorescence in the dark (F_m_ – minimal fluorescence in the dark, F_o_). Mean values ± standard deviations, *n* = 3; analyzed statistically via linear regression.

**FIGURE 10:**
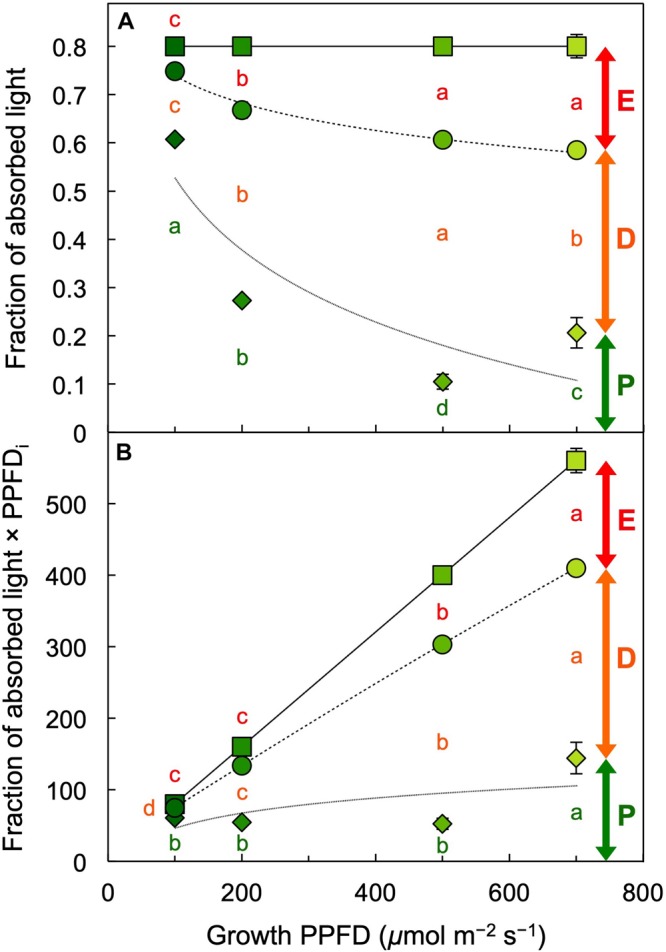
**(A)** Fraction of absorbed light utilized in photochemistry (P, green span labeled for 700 μmol photons m^–2^ s^–1^), thermally dissipated (D, orange span labeled for 700 μmol photons m^–2^ s^–1^), and in excess (E, red span labeled for 700 μmol photons m^–2^ s^–1^) and **(B)** the product of the latter fractions and photosynthetic photon flux density incident (PPFD_i_) upon *L. gibba* fronds as a function of growth PPFD. See the section “Materials and Methods” and Demmig-Adams et al. (1996a) for calculations. Mean values ± standard deviations, *n* = 3 for all growth PPFDs; different lower-case letters signify statistical differences at *P* < 0.05 via one-way analysis of variance and *post hoc* Tukey–Kramer HSD test.

The photochemical efficiency of those PSII centers that remained open at each growth PPFD (F_v_′/F_m_′) declined with increasing growth PPFD (Figure 8A), which indicates increasing removal of excess excitation energy by photoprotective thermal energy dissipation (see Figure 3). The actual fraction of PSII centers that are closed (reduced) under each growth PPFD, 1 – q_P_, increased with increasing growth PPFD up to 500 μmol photons m^–2^ s^–1^, but did not increase further under 700 μmol photons m^–2^ s^–1^ (Figure 8B). Photochemical efficiency at the actual percentage of closed PSII centers under each respective growth PPFD (F_v_′/F_m_′ × q_P_) decreased strongly with increasing growth PPFD (Figure 8B). Potential maximal PSII photochemical efficiency (F_v_/F_m_) of fronds darkened for 5 min immediately upon removal from growth PPFD (− Recovery) exhibited a moderate decline as a function of growth PPFD, but rebounded quickly over 30 min of recovery (+ Recovery) in low light (Figure 8C). The resulting high dark F_v_/F_m_ levels in *L. gibba* after this brief recovery period indicate an absence of photoinhibitory inactivation of photochemistry, which is consistent with *L. gibba*’s continuously high growth rates (Figures 2, 4). Figure 9 shows that dark F_v_/F_m_ increased in proportion to removal of zeaxanthin (expressed as zeaxanthin level relative to the total xanthophyll cycle pool), indicating that the residual minor depression of dark F_v_/F_m_ after 30 min of recovery is due to some sustained thermal energy dissipation. Since this residual minor depression in dark F_v_/F_m_ precludes quantification of energy dissipation from the degree of quenching of maximal fluorescence in light versus darkness (Adams and Demmig-Adams, 2004; Adams et al., 2013, 2014; Logan et al., 2014), alternative approaches (see Figure 3 and section “Materials and Methods”) were used to estimate energy dissipation activity (Figure 10).

Figure 10 provides a comprehensive accounting of absorbed photons and their pathways in the photochemical system of *L. gibba* as a function of growth PPFD. The percentage of absorbed photons utilized in photochemistry (P) was highest in fronds grown under 100 μmol photons m^–2^ s^–1^ and declined with increasing growth PPFD (P in Figure 10A), which is consistent with the pronounced decline in light-use efficiency of frond area production as a function of growth PPFD (Figure 4C). In the absence of photoinhibitory inactivation of photosynthesis in these plants, the fraction of absorbed photons dissipated in photoprotective thermal energy dissipation (D) under each respective growth PPFD can be assessed as 0.8 – F_v_′/F_m_′, which increased strongly and then plateaued with increasing growth PPFD (D in Figure 10A). The fraction of absorbed photons used neither in photochemistry nor dissipated thermally (E for Excess), F_v_′/F_m_′ × (1 – q_P_), increased only modestly and then also plateaued (E in Figure 10A).

In contrast to the fraction of photons, the actual number of photons entering the three possible pathways continues to increase with increasing absorption of light under increasing growth PPFD. Multiplication of each fraction of absorbed photons × the total number of photons absorbed at each growth PPFD would provide numbers of photons entering each pathway (P, D, and E). The total number of absorbed photons is, however, not available since absorptance measurements in these fronds could not separate photons into those absorbed by chlorophyll versus those absorbed by the yellow (carotenoid) pigments that do not transfer photons to photochemical reaction centers. To illustrate the concept of increasing rates of P, D, and E with increasing growth PPFD, Figure 10B shows the product of incident PPFD (PPFD_i_) × the fractions of absorbed photons going into pathways P, D, and E from Figure 10A. The declining chlorophyll *a* + *b* levels with increasing growth PPFD (Figures 2, 5A) presumably cause photon absorption to deviate increasingly from incident PPFD, and true rates of P, D, and E will thus be proportionally lower. Nevertheless, the estimated rate of thermal energy dissipation shown in Figure 10B provides the expected match for the level of the dissipater zeaxanthin on a chlorophyll basis (Figure 6A), with both parameters exhibiting continued increases with increasing growth PPFD. This result is also consistent with the close correlation between zeaxanthin level and dark F_v_/F_m_ (Figure 9) subsequent to removal from growth PPFD.

## Discussion

This study addressed the impact of growth light intensity on plant growth and photosynthesis as well as on the concentrations of photosynthetic pigments with nutritional value for the human consumer. We focused on select vitamins and other essential antioxidant metabolites with well-documented health benefits that are (i) diet-derived nutrients for the human consumer, (ii) act synergistically with each other in both plants and animals, and (iii) are upregulated in plants in response to environmental cues (Grace and Logan, 1996; Logan et al., 1996, 1998a,b; Demmig-Adams and Adams, 2002; Adams et al., 2016). Humans require a number of antioxidant metabolites that cannot be synthesized in the human body and must be supplied by the diet, preferably via consumption of whole foods. Future studies should address the effect of growth light intensity on additional antioxidants with relevance to human nutrition, such as vitamins C and E, and phenolics.

### Maintenance of Similar Growth Rates Over an Extreme Range of Light Environments

The results reported here demonstrate that *L. gibba* is able to achieve a remarkably high growth rate and light-use efficiency of plant growth under low growth light intensity. *Lemna gibba* achieves this high growth rate by superior light absorption – due to a combination of apparent minimal self-shading in the arrangement of its leaves and high chlorophyll levels under low light. The low levels of self-shading in the thin *L. gibba* leaves represent a natural adaptation that favors high light-use efficiency and is an alternative to current efforts to engineer crops with truncated chlorophyll antennae by way of reduced levels of outer chlorophyll antennae Lhcb (Ort et al., 2015; Kirst et al., 2017, 2018; Song et al., 2017).

Conversely, *L. gibba*’s growth rate also remained remarkably high even under a growth PPFD that supplied a total amount of photons per day similar to that received on the brightest and longest day of the year on Earth. One can consider this maintenance of high growth rates as evidence for a high degree of plant phenotypic plasticity, or robustness, of *L. gibba* with respect to extreme variation in the environment. It should be noted that *L. gibba* maintained these high growth rates under very high growth PPFDs despite the minimal self-shading in its relatively thin leaves. Self-shading in thick, high-light-grown leaves, as well as the overlapping nature of leaves in the canopy, of other plant species offers some structural photoprotection against excess light. The ability of *L. gibba* to avoid photoinhibition of photosynthesis under high growth PPFDs despite possessing thin leaves is thus a remarkable quality, which is apparently based on a combination of strong downregulation of chlorophyll synthesis and strongly increased biochemical photoprotection that kept excess excitation energy from building up beyond a modest level. This pronounced biochemical photoprotection included high levels of thermal dissipation of excitation energy, accumulation and retention of zeaxanthin in the amount of up to a third of the xanthophyll cycle pool at the highest growth PPFD for a period of 30 min in low PPFD, and accumulation of lutein. García-Plazaola et al. (2002) likewise found that *L. minor* strongly upregulated the pool of xanthophyll cycle pigments when grown under higher versus low PPFD and synthesized additional zeaxanthin when transferred to an even higher PPFD for several hours. The ability to sustain high area growth over a wide range of light environments is presumably advantageous for a floating plant like duckweed that occurs both in open ponds and areas around the edge of ponds that may be shaded by emergent macrophytes (e.g., cattails, sedges), overhanging terrestrial vegetation (e.g., willows, cottonwoods), or man-made structures (docks, bridges, etc.). Rapid coverage, and shading, of a pond by duckweed presumably serves to discourage the growth of algae that compete for nutrients.

### High Phenotypic Plasticity With Respect to Highly Excessive Light

Lutein is abundant in all leaves even under low growth PPFDs because it is not a competitor with photochemistry; lutein detoxifies a triplet excited state of chlorophyll not used for photochemistry (Dall’Osto et al., 2006). In contrast, zeaxanthin is a direct competitor for photochemistry and de-excites the same singlet excited state of chlorophyll used for photochemistry (Demmig-Adams and Adams, 1992; Park et al., 2018, 2019). Several regulatory mechanisms ensure that zeaxanthin is formed in leaves only under excess light and is typically removed quickly upon return to non-excessive light levels (Demmig-Adams et al., 1996b).

The declining chlorophyll content and increase in the proportion of individual carotenoids relative to chlorophyll at high growth PPFD were consistent with some downregulation of the outer chlorophyll light-harvesting complex that maximizes light absorption in low light (Lhcb; the major Chl [*a*+*b*]- and lutein-binding light-harvesting complex) and with the role of lutein in Chl triplet de-excitation in Lhcb (Dall’Osto et al., 2006) as well as some downregulation of photosystem-core antenna complexes (that bind β-carotene). The strong accumulation of zeaxanthin despite the decline in chlorophyll level is consistent with localization of important zeaxanthin-binding sites with roles in thermal energy dissipation (Park et al., 2018, 2019) in linker proteins between photosystems and their outer Lhcb complexes. Under the highest growth PPFDs, additional zeaxanthin may be located in the lipid fraction of the photosynthetic membrane, where it can contribute to direct protection of membrane lipids (Havaux and García-Plazaola, 2014) in synergistic interaction with vitamin E (Wrona et al., 2003, 2004; Schneider, 2005). The findings of the present study thus confirm that zeaxanthin production in *L. gibba* requires very high growth PPFDs, while production of both lutein and β-carotene on a frond area basis declined somewhat at the highest growth PPFDs used here. β-carotene can serve as an antioxidant in both plants (Telfer, 2014) and animals (Stahl and Sies, 2012) and is, furthermore, the precursor (pro-vitamin A) in the synthesis of vitamin A, the precursor of the light-absorbing (retinal) component of vision purple in the human eye (Polutchko et al., 2015).

Coupling of chlorophyll downregulation and upregulation of zeaxanthin-associated photoprotection by *L. gibba* under excess levels of light combines two mechanisms with different features. The downregulation of chlorophyll content under high growth PPFDs cannot be reversed on short time scales of minutes to hours. However, rapidly reversible, thermal dissipation of excess excitation offers a high degree of flexibility with simultaneous strong photoprotection and a quick resumption of high light-use efficiency upon return to limiting light. The combination of these two biochemical adjustments – modulation of light-harvesting capacity/chlorophyll content and modulation of carotenoid levels and energy dissipation (assessed from F_v_′/F_m_′) – was apparently potent enough to (i) effectively limit build-up of excess excitation as well as prevent PSII centers from closing entirely even at the highest growth PPFDs and (ii) preserve the ability to quickly return to high PSII efficiency upon transfer to low light.

The concomitant changes in zeaxanthin content (and xanthophyll cycle conversion state to zeaxanthin) with changes in PSII efficiency are consistent with the well-documented role of zeaxanthin in thermal dissipation of excess absorbed light (Demmig-Adams and Adams, 1996, 2006; Demmig-Adams et al., 2012, 2014; Park et al., 2018, 2019). Theory predicts that the relationship between the absolute zeaxanthin level and the fraction of absorbed photons dissipated by zeaxanthin as thermal energy is curvilinear since the fraction of absorbed photons dissipated thermally is constrained to a maximum of 80% of absorbed photons. In contrast, zeaxanthin level and the rate constant of thermal dissipation both increase linearly with the absolute amount of photons dissipated thermally (Demmig-Adams et al., 1989a, b). A linear relationship between the rate constant of thermal energy dissipation and zeaxanthin level on a dry mass basis was empirically observed in leaves of several plant species by Demmig-Adams et al. (1989a, b).

### Source-Sink Balance in Duckweed

Duckweed’s storage capacity for photosynthetically produced sugars is presumably limited by the virtual absence of non-green parts that can serve as major sinks for sugars in other plant species (Adams et al., 2014, 2018). This absence of non-green parts is what makes duckweed near-100% edible. Furthermore, this scenario may be the reason for duckweed’s prolific growth of new fronds as the species’ sole or main sink for sugars and the associated fast area growth and doubling times of duckweed species (Sree et al., 2015; Ziegler et al., 2015). The finding that maximal photosynthetic capacity did not increase with increasing growth PPFD between 100 and 700 μmol photons m^–2^ s^–1^ is consistent with sink limitation and starch accumulation as factors counteracting photosynthetic upregulation as part of a feedback loop that regulates photosynthesis by the demand for carbohydrate from the rest of the plant (Adams et al., 2013, 2014, 2018; Demmig-Adams et al., 2017). However, it is noteworthy that, despite the accumulation of starch, there was neither downregulation of photosynthetic capacity (see also McLaren and Smith, 1976) nor any decrease in area growth rate with increasing growth PPFD up to the highest growth PPFD. As stated above, high area growth rates are presumably ecologically advantageous for a floating plant like duckweed that benefits from an ability to rapidly cover large areas of water.

### The Next Step in Duckweed Agriculture for Space Exploration

Compared to the light levels needed for remarkably high rates of *L. gibba* photosynthesis and growth, much higher light levels are required for strong induction of the essential antioxidant zeaxanthin. A similar relationship has been demonstrated in many other plant species for this relationship between light level needed to saturate photosynthesis and induction of strong zeaxanthin formation (see, e.g., Demmig-Adams et al., 1989a) as well as induction of strong accumulation of the antioxidant vitamins C and E and the enzymatic antioxidant glutathione (Grace and Logan, 1996; Logan et al., 1996). Excessively high growth light intensity (see Seginer et al., 2006 for lettuce) or other environmental stressors, on the other hand, can drive up excitation pressure to a level that causes growth reductions and photoinhibition (see, e.g., Adams et al., 2013) and even antioxidant destruction by light stress (see Havaux and García-Plazaola, 2014). Therefore, the challenge in designing light protocols for simultaneous optimization of plant yield, nutritional quality, and resource-use efficiency is that light-use efficiency of plant growth is maximal under low light input, while production of zeaxanthin and other essential antioxidants requires high light. In applications where the cost of light-energy input is not an issue, plants could be grown under high PPFD. However, if light input is an issue, the input of 600% more light to attain a 25% greater relative growth rate (as seen at 700 versus 100 μmol photons m^–2^ s^–1^) may not be justifiable.

Insight from plant ecophysiology can offer solutions for co-optimization of plant yield, energy-use efficiency, and nutritional quality. We discovered (Adams et al., 1999) that plants growing in the shaded understorey of a forest periodically punctuated by shafts of full sunlight (sunflecks penetrating the canopy) produce and continuously retain considerable amounts of zeaxanthin. Based on this finding, we used a combination of low background light intensity and a few high-light pulses in climate-controlled growth chambers to simultaneously keep energy-use efficiency high and yet produce and retain zeaxanthin under low background light. We obtained proof-of-concept for the validity of this approach with the model species *Arabidopsis thaliana* (Cohu et al., 2014). The latter study offered the conclusion that “growth light environment … can be exploited to simultaneously optimize nutritional quality … as well as biomass production of leafy greens suitable as bioregenerative systems for long-duration manned spaceflight missions.” It may thus be possible to minimize, or circumvent altogether, the trade-off between light-use efficiency of growth and nutritional quality with this novel growth protocol to co-optimize all desired features.

## Data Availability Statement

The data presented in this manuscript are available from the corresponding author upon reasonable request.

## Author Contributions

BD-A, CE, and WA wrote the grant on which this study is based. JS carried out the experiments, biochemical assays, and statistical analyses with contributions from CE, BD-A, ML-P, and WA. BD-A wrote the manuscript with contributions from WA, JS, and CE.

## Conflict of Interest

CE has financial interest in Space Lab Technologies, LLC, a company that may be affected by this research. A Memorandum of Understanding approved by the University of Colorado manages potential conflicts arising from this relationship.

The remaining authors declare that the research was conducted in the absence of any commercial or financial relationships that could be construed as a potential conflict of interest.

## Footnotes

1 http://www.ruduckweed.org
